# Contextual fear response is modulated by M-type K+ channels and is associated with subtle structural changes of the axon initial segment in hippocampal GABAergic neurons

**DOI:** 10.3934/Neuroscience.2023003

**Published:** 2023-03-29

**Authors:** Sara Arciniegas Ruiz, Eliav Tikochinsky, Vardit Rubovitch, Chaim G Pick, Bernard Attali

**Affiliations:** 1 Department of Physiology and Pharmacology, Sackler School of Medicine, Tel Aviv University, Tel Aviv, Israel; 2 Sagol School of Neuroscience, Sackler School of Medicine, Tel Aviv University, Tel Aviv, Israel.; 3 Department of Anatomy and Anthropology, Sackler School of Medicine, Tel Aviv University, Tel Aviv, Israel

**Keywords:** fear conditioning, GABAergic neurons, hippocampus, neuronal plasticity, potassium channels

## Abstract

**Background:**

In the fear memory network, the hippocampus modulates contextual aspects of fear learning while mutual connections between the amygdala and the medial prefrontal cortex are widely involved in fear extinction. G-protein-coupled receptors (GPCRs) are involved in the regulation of fear and anxiety, so the regulation of GPCRs in fear signaling pathways can modulate the mechanisms of fear memory acquisition, consolidation and extinction. Various studies suggested a role of M-type K+ channels in modulating fear expression and extinction, although conflicting data prevented drawing of clear conclusions. In the present work, we examined the impact of M-type K+ channel blockade or activation on contextual fear acquisition and extinction. In addition, regarding the pivotal role of the hippocampus in contextual fear conditioning (CFC) and the involvement of the axon initial segment (AIS) in neuronal plasticity, we investigated whether structural alterations of the AIS in hippocampal neurons occurred during contextual fear memory acquisition and short-time extinction in mice in a behaviorally relevant context.

**Results:**

When a single systemic injection of the M-channel blocker XE991 (2 mg/kg, IP) was carried out 15 minutes before the foot shock session, fear expression was significantly reduced. Expression of c-Fos was increased following CFC, mostly in GABAergic neurons at day 1 and day 2 post-fear training in CA1 and dentate gyrus hippocampal regions. A significantly longer AIS segment was observed in GABAergic neurons of the CA1 hippocampal region at day 2.

**Conclusions:**

Our results underscore the role of M-type K + channels in CFC and the importance of hippocampal GABAergic neurons in fear expression.

## 1. Introduction

The neural circuits underlying the acquisition, retention and retrieval of contextual fear have been well characterized in rodents [1]–[3]. The acquisition and retrieval of contextual fear memory involves coordinated neural activity of the hippocampus, medial prefrontal cortex (mPFC), and amygdala [4]–[9]. The contextual information encoded in the hippocampus is conveyed to the mPFC and amygdala for contextual fear acquisition. Contextual fear conditioning (CFC) is a naturally occurring phenomenon whereby a particular context becomes feared. In experimental fear conditioning, a contextual conditioned stimulus (CS) is usually paired with a mild foot shock unconditioned stimulus (US). After establishment of conditioned fear, the CS alone can induce conditioned fear responses such as freezing. Recurrent expositions of the CS without US can reduce the freezing response, inducing attenuation of the fear component associated to the traumatic memory and is defined as fear extinction [10]–[12]. Fear extinction is a form of inhibitory learning [13],[14]. However, there also is indication that extinction could be an “unlearning” process resulting to de-potentiation of potentiated synapses within the amygdala [15]–[17].

Fear memories depend on the interplay between the basolateral amygdala (BLA), the hippocampus and the mPFC. In the fear memory network, the hippocampus modulates contextual aspects of fear learning [18] while mutual connections between the amygdala and mPFC are widely involved in fear extinction [19]. Recent lines of evidence indicate that contextual fear memories appear to be consolidated and maintained by both amygdala and hippocampus [9]. With regard to brain functions, the hippocampus has been shown to be one of the most critical brain regions in contextual fear acquisition, which significantly interacts with the mPFC during the contextual associations in healthy individuals [10]. Defects in the mechanisms underlying fear extinction are believed to be the basis of some anxiety and trauma-related disorders [20].

Neuromodulators released during and after a fearful experience promote the consolidation of fear memory for that experience. In addition, G-protein-coupled receptors (GPCRs) play an important role in the regulation of these neurotransmitters signaling pathways; hence, GPCRs can modulate the mechanisms of fear memory acquisition, consolidation and extinction [21]. Several lines of evidence indicate that the activity of infralimbic prefrontal cortex (IL) is critical for inhibiting inappropriate fear responses following extinction learning [22]. Disruption of IL function by using electrolytic lesions or pharmacological inactivation before extinction training impaired subsequent recall of extinction memory [23]–[25]. It was also shown that fear conditioning and extinction alter the intrinsic excitability and bursting of IL pyramidal neurons in brain slices [26]. Interestingly, pharmacological enhancement of IL excitability and bursting by infusing into IL the M-type K+ channel blocker XE991, before extinction training reduced fear expression and facilitates extinction in fear-conditioned rats [27]. Conversely, reducing IL excitability and bursting with the M-type K+ channel agonist, flupirtine, had the opposite effect, suggesting that the intrinsic excitability and bursting of IL neurons regulate fear expression even before extinction [27]. More recently, another study showed that injection of the M-type K+ channel blocker XE991 immediately after moderate fear conditioning caused a dose-dependent enhancement of fear consolidation [28], suggesting that inhibition of the M-current increases the strength of fear memory consolidation, a conclusion that is in dissonance with the former study [27]. In this context, the role of M-type K+ channels in fear conditioning and their pharmacological modulation to facilitate fear extinction need to be clarified yet.

In the present study, we examined the impact of M-type K+ channel blockade or activation on contextual fear acquisition and retention of the fear experience in a short-interval extinction training. In addition, regarding the pivotal role of the hippocampus in contextual fear conditioning, we investigated whether structural alterations of the axon initial segment (AIS) of hippocampal neurons occurred during contextual fear memory acquisition and retention.

## 2. Materials and methods

### 2.1. Animals

Male C57BL6/J mice (7 weeks old, 22 ± 5 g, ENVIGO Israel- https://www.envigo.com/) were randomly housed in groups of four during 7 days in reverse cycle prior to experiments and provided with food and water ad libitum. Housing was maintained at constant temperature (21 ± 1°C) and humidity (55 ± 10%) under a 12 hours' light/dark cycle (8 am–8 pm). All studies were performed during the animal's dark phase. Mice were randomly allocated in experimental groups. All experimental protocols conformed to the guidelines of the Institutional Animal Care and Use Committee of Tel-Aviv University (Nº 01-018-088), Israel and to the NIH (National Institutes of Health) guidelines.

### 2.2. Contextual fear-conditioning procedures

The apparatus and general procedures for contextual fear conditioning (CFC) consisted of a Ugo Basile, ANY-maze controlled Fear Conditioning System (Fear Conditioning 2.1), which entailed a sound-attenuating box, with ventilating fan, a dual (visible/I.R.) light and a USB-camera. Each unit has an individual controller on-board. Mice were tested in a conditioning chamber (17 × 17 × 25(h) cm), containing a stainless-steel shock-grid floor. The detection of freezing is automated and based on video analysis using the Ethovision XT software®. The context consisted in black and white squares decorated walls in the cages.

### 2.3. Contextual fear conditioning experiments

Each experiment consisted of three phases: habituation for context, fear conditioning, and testing. In the first day, animals were placed inside the context chamber during 5 min, where they were placed during 2 min for a habituation period and in the remaining 3 min, they received three non-signaled foot shocks (2-sec duration, 0.5 mA, at 1 min apart). 24 hours later, the test was conducted, where mice were placed back into the conditioning context without the foot shocks for 3 min. Freezing behavior (defined as a complete absence of movement, except for respiration) [29] was measured using automated procedures [30]. The apparatus was cleaned each time between subjects with Virusolve® + (Amity International).

### 2.4. Contextual Re-exposure procedure (CRP, short-time extinction training)

After CFC, animals were placed inside the same chamber with the same context during 3 min without the foot shocks. This procedure was repeated at 48, 72, and 96 hours after the fear conditioning. During each of these days, freezing behavior was evaluated and compared with the freezing response on the first test day, day 1.

### 2.5. Drug administration

The drugs used were: XE991 dihydrochloride (4-pyridinylmethyl-9(10H)-anthracenone, Tocris Bioscience, UK) and retigabine dihydrochloride (Alomone labs, Israel). Animals were habituated to be handled and to receive injections of saline solution for three days before fear conditioning. Animals were injected intraperitoneally with 150 µl of vehicle (saline), once a day at the same hour for three days. Then, the CFC was performed as described above. A Group of animals received immediately after the foot shock session a single injection of XE991 (1 mg/kg), retigabine (RTG, 4 mg/kg) or vehicle (saline), based in previous studies [28]. Another group of animals received a single injection of XE991 (2 mg/kg), retigabine (10 mg/kg) or vehicle 15 min before the CFC, based on a dose-response pre-trial performed previously. The volume of all the drugs was adjusted to 150 µl. The CRP was performed as described above.

### 2.6. Experimental design

*CFC Experiment 1*. Forty animals were used for CFC and CRP to obtain samples for immunohistochemistry (IHC). Animals were randomly divided into control group and 4 experimental groups in equal amount (n = 8): control (context, but did not receive footshocks), and experimental groups (context, and footshocks) were named as day 1, day 2, day 3, and day 4. All mice were placed in the chamber with context for CFC, as previously described, but animals of control group did not receive shocks; 24 hours later, all mice were placed back into the conditioning context without the foot-shocks, and freezing behavior was tested. One hour after the test, control group and day 1 group were euthanized and brains were removed for IHC. The rest of animals were kept and used for the CRP procedures. One hour after the test, each day of the procedure, a group of animals was euthanized and brains removed for IHC.

*CFC Experiment 2*. For the drug administration procedure, two groups of animals were used to receive the drugs before or after CFC in the next way: 1) initially a group of 120 animals was used to receive the drugs after the foot-shocks. Animals were randomly divided in 3 experimental subgroups in equal amounts (n = 40): retigabine, XE991 and vehicle (saline). 2) A second group of 90 animals was used for the drug administration before the foot-shocks. Animals were randomly divided in 3 experimental subgroups in equal amount (n = 30): retigabine, XE991 and vehicle (saline). CFC and CRP were performed as described above.

### 2.7. Immunohistochemistry

One hour after CFC, mice were deeply anesthetized using a chamber filled gradually with carbone dioxide until unconsciousness [31], then the animals were transcardially perfused with 4% paraformaldehyde (EMS Ban-naor LTD, Israel) followed by PBS (Hylabs, Israel). The whole brain was removed and kept in 4% paraformaldehyde; 24 hours later, the solution was changed to 1% paraformaldehyde and maintained until cutting. Previous to cutting, brains were cryopreserved in 0.1 M PBS containing 30% sucrose (Bio-Lab ltd, Israel) for 48 h [32]. Two hours before cutting, brains were drained, dried, and frozen at −4 °C. Brains were serially sectioned at 35 µm width in a coronal orientation using a Leica CM 1850 cryostat (Leica Microsystems, Buffalo Grove, IL). The brain sections were processed for free-floating immunohistochemistry [33] using a cryo-protectant solution composed of glycerin (Bio-Lab ltd, Israel), ethylene glycol (Bio-Lab ltd, Israel) and PBS (Hylabs, Israel). The free-floating method consisted of:

Day 1: tissue sections were washed three times with phosphate buffer PBS, 5 min each. Then, samples were incubated in 10 mM tri-sodium citrate solution pH 8.5 (Emsure®, Merck, Germany), during 30 min at 80 °C for antigen retrieval, and then cooled down to room temperature. Then, slices were washed extensively with PBS and PBST [PBS + 0.1% Triton X-100 (Sigma Aldrich, St Louis, MO, USA)] and incubated with a blocking buffer consisting of 10 % Goat Serum (Sigma Aldrich, St Louis, MO, USA) in PBST for 1 hour. This was followed by an incubation for 48 hours at 4 °C with primary antibodies in PBST containing 2% goat serum. Two different stainings were performed with the slices; the primary antibodies used for the first staining were: mouse monoclonal antibody anti-Ankyrin G (1:300, Antibodies Incorporated, catalog N^o^: 75146, RRID: AB_10673030), Rabbit polyclonal antibody anti-MAP2 (1:800, Synaptic Systems, catalog N^o^: 188002, RRID: AB_2138183), and polyclonal antibody guinea pig Anti-c-Fos (1:800, Synaptic Systems, catalog N^o^: 226004, RRID: AB_2619946); the primary antibodies used for the second staining were: polyclonal antibody rabbit anti-vGAT (1:400, Synaptic Systems, catalog N^o^: 131002, RRID:AB_887871 ), monoclonal antibody mouse anti-vGLUT1 (1:1000, Synaptic Systems, catalog N^o^: 135511, RRID:AB_887879), and polyclonal antibody guinea pig Anti-c-Fos (1:800, Synaptic Systems, catalog N^o^: 226004, RRID: AB_2619946). Anti-MAP2 antibodies were used to identify cellular morphology and distribution as described previously [7],[11],[34],[35].

Day 2: tissue sections were washed five times with PBST, and then incubated with the appropriate secondary antibody for 1h in a PBTS solution with 2% goat serum in dark room. The following isotype-specific secondary antibodies were used: Alexa Fluor 488-conjugated donkey anti-mouse IgG (H+L), (Jackson Immunoresearch laboratories Inc., USA); Alexa Fluor 633-conjugated goat anti-guinea pig IgG (H+L), (Invitrogen, ThermoFisher scientific, USA) and Cy3 donkey anti-rabbit IgG (H+L), (Jackson Immunoresearch laboratories Inc., USA). After secondary antibody incubation, tissue sections were washed two more times with PBS, then mounted on glass microscope slides (Histobond +, Marienfeld, Germany) with aqueous mounting medium (Fluoromount®, Sigma Aldrich, St Louis, MO, USA) and covered with glass coverslips.

### 2.8. Confocal Microscopy

Fluorescent images were acquired using a Leica TCS SP5 Confocal Laser Scanning Microscope with a Plan-Apochromat objective (40x/1.2 Oil immersion). Multitrack acquisition was performed with scanning speed of 400 Hz, frame size of 1024 × 1024 pixels, X phase correction of −30, and same pinhole setting for all three channels. The images were obtained from consecutive sections containing the hippocampus (8–12 sections per mouse) in both hemispheres. All confocal images were blindly processed using Image J US NIH (http://imagej.nih.gov/ij). The total amount of c-Fos positive cells was obtained, and subsequently discriminated according to their co-immunolocalization with vGAT or vGLUT, and used as reference for localization of ANKG: length of AIS, and distance from soma (MAP2) was measured. The total amount of slices processed were: 71–89 for c-Fos counting, in CA1 region 85–108 were used for measurements of ANKG vGAT cells, and 39–55 in vGLUT cells, in DG region 74–98 were processed for measurements of ANKG in vGAT cells, and 14–59 in vGLUT cells. All neuronal measurements were grouped by animal for further statistical analysis.

### 2.9. Statistical analysis

All data were expressed as means ± SEM. Statistical analysis was performed using SPSS statistics 20 (IBM, USA) and GraphPad PRISM 9.1 (GraphPad Software LLC). Data normality was determined by the Shapiro-Wilks test and the homogeneity of variances by the Levene test. Data were analyzed using one-way or two-way ANOVA test to determine differences between the means in all independent groups. *Post hoc* tests were performed using Dunnett's test to compare the experimental groups versus the control, or Tukey test or Fisher LSD test to compare all the experimental groups between them, and identify similarities or patterns. Statistically significant differences were reported when p < 0.05.

## 3. Results

### 3.1. Contextual Fear Conditioning

**Figure 1.**
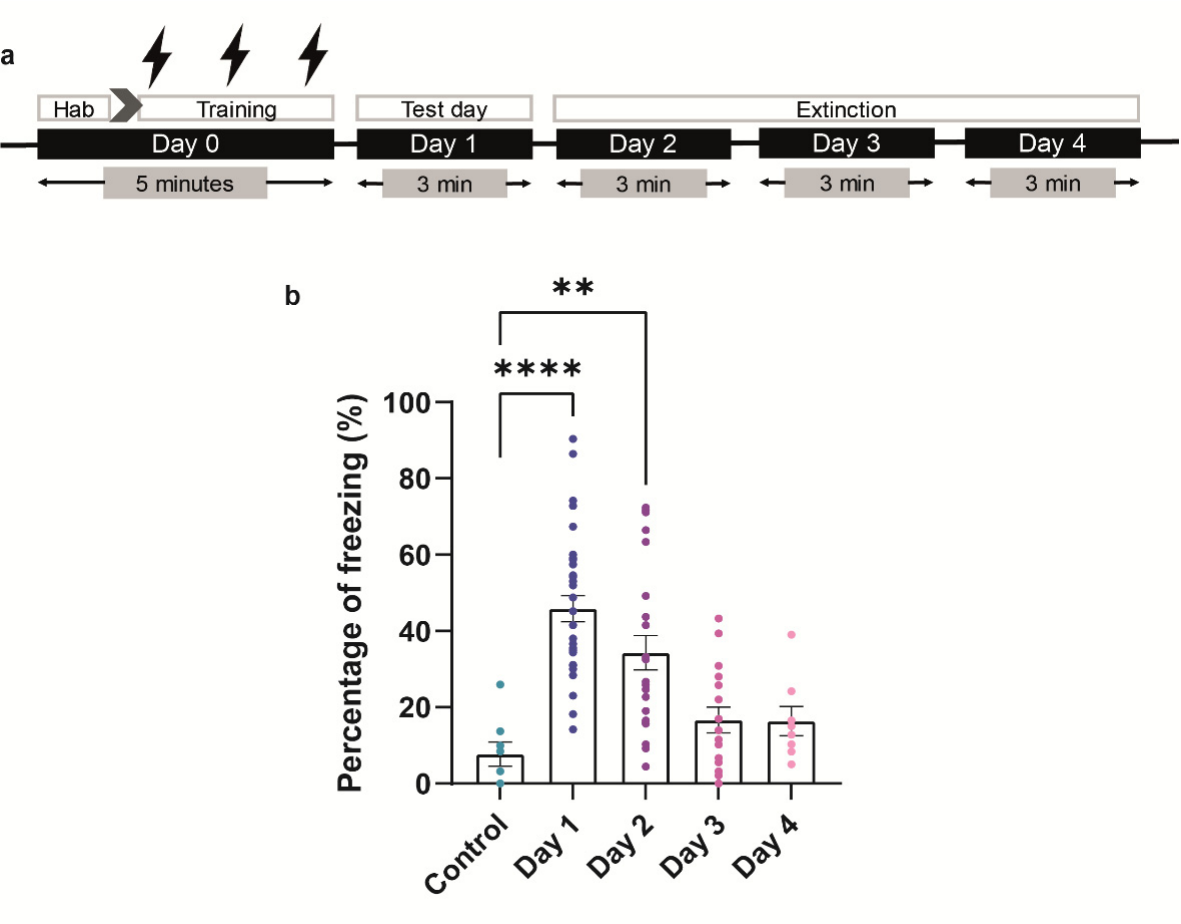
Contextual Fear conditioning. (a) Protocol of Contextual Fear conditioning. Day 0 is referred to the training day: after a habituation period of 2 min, animals received 3 foot shocks, each lasting 2 sec at one min interval. (b) Behavioral profile showing fear expression at day 1 and subsequent re-exposure to the context at days 2–4. No shocked animals are referred to control. Data are expressed as mean ± SEM. The total number of mice was 40, where each day a number of animals were sacrificed for IHC processing. Animals were randomly divided in control group and 4 experimental groups for sample collection. Control (n = 8), day 1 (n = 32), day 2 (n = 24), day 3 (n = 16) and day 4 (n = 8). ANOVA, F(4,82) = 13.2; *p < 0.05, **p < 0.01, ***p < 0.001, ****p < 0.0001 with Dunnett's post-hoc test.

Mice were trained to associate a context (CS) with a painful unconditioned stimulus (a mild electric foot shock, US). After training, mice (CS+US) showed on day 1 an average freezing of 45.86 ± 3.398 % (n = 32) during the test session as compared to the control group (CS without US), which froze 7.708 ± 3.178 % (n = 8) (Fig. 1; ANOVA, F(4,82) = 13.2; p < 0.001). **p < 0.01, ***p < 0.001 by Post Hoc Dunnett's test). During the CRP (Contextual Re-exposure Procedure) period, though significantly larger than control, a decrease in freezing of 34.3 ± 4.5 % (n = 23) was observed at day 2, whereas at day 3 and day 4 freezing declined to values not significantly different from the control group (16.67 ± 3.373 % (n = 16) and 16.4 ± 3.822 % (n = 8), respectively; Fig. 1).

### 3.2. Regulation of the contextual fear response by pharmacological modulation of M-type K+ channels

**Figure 2.**
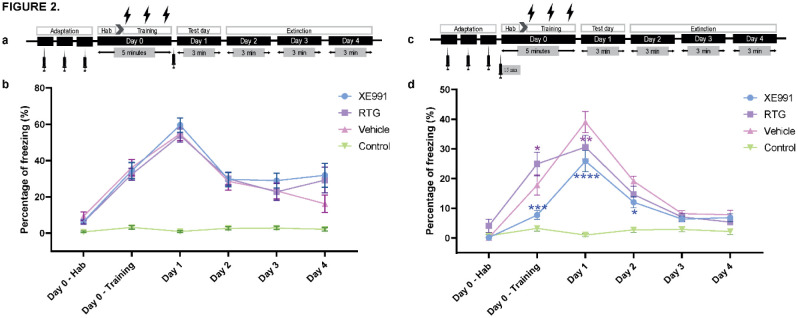
Regulation of the contextual fear response by pharmacological modulation of M-type K+ channels. (a and c) Protocol of contextual fear conditioning upon single intraperitoneal (IP) drug injections of XE991, retigabine (RTG) and vehicle (sterile water). All animals underwent an adaptation training for injection handling, where animals received single IP injections of vehicle during 3 consecutive days, before the training session. In (a), animals received IP drug injection immediately after the training session. In (c), animals received IP drug injection 15 min before the training session. (b) Behavioral profile of the CFC when IP drug injection was carried out immediately after the foot shocks. Single IP injections were as follows: XE991 (1 mg/Kg), retigabine (4 mg/Kg) and vehicle in a total volume of 150 µL. No shock (n = 12 for each day from day 0 to day 4), for XE991, retigabine and vehicle groups (for each group n = 40 at day 0 and day 1, n = 30 at day 2, n = 20 at day 3 and n = 10 at day 4). Data are expressed as mean ± SEM. No statistical differences were found for any treatment in the shocked groups vs. vehicle (p > 0.05). *Two-way ANOVA* by treatments *F(3, 589)= 40.16, p < 0.0001*, and by days *F(5, 589) = 35.63, p < 0.0001*. Multiple comparisons by Fisher LSD's post-hoc test (vs. vehicle group): *p < 0.05, **p < 0.01, ***p < 0.001, ****p < 0.0001. (d) Behavioral profile of the CFC when IP drug injection was carried out immediately before the training session. Single IP injections were as follows: XE991 (2 mg/Kg), retigabine (10 mg/Kg) and vehicle in a total volume of 150 µL. No shock (n = 12 for each day from day 0 to day 4), for XE991, retigabine and vehicle groups (each group n = 30 for each day from day 0 to day 4). Data are expressed as mean ± SEM. There is a statistical difference between the XE991 and the vehicle groups. Two-way ANOVA by treatments *F(3, 554) = 26.3, p < 0.0001*, and by days *F(5, 554) = 41.01, p < 0.0001*. Multiple comparisons by Fisher LSD's post-hoc test (vs. vehicle group): *p < 0.05, **p < 0.01, ***p < 0.001, ****p < 0.0001.

To clarify the role of M-channels in CFC, we first performed an experiment using the same doses described previously [28] with a single systemic injection of the M-channel blocker XE991 (intraperitoneal 1 mg/kg) or the M-channel opener retigabine (intraperitoneal 4 mg/kg) immediately after the foot shock session (Fig. 2a, b). Our results show that is no statistical differences in the freezing response in the shocked mice between both drugs and the vehicle at any day (*n = 40*, *p > 0.05*), (Fig. 2a, b). However, freezing response from all the treatments statistically differed from the no-shocked group in all the days, except from the habituation (*two-way ANOVA, F (3, 589) = 40.16, p < 0.0001)*. The post-hoc test revealed that in day 4 the no-shocked group differed from either drugs treatments (*p < 0.001*), but not with vehicle (*p > 0.05*). Next, we tried to carry out a single systemic injection of the M-channel blocker XE991 (intraperitoneal 2 mg/kg) or the M-channel opener retigabine (intraperitoneal 10 mg/kg) 15 minutes before the foot shock session (Fig. 2c, d). XE991 showed significant reduction of the freezing even during the training session (*n = 30; two-way ANOVA F(3, 554) = 26.31, p < 0.0001*). The post-hoc analysis showed significant reduction of the freezing at Day 1 (*p < 0,0001*) and Day 2 (*p = 0.019*). In fact, XE991 reduced fear expression and facilitated recovery (Fig. 2c, d). On the other hand, retigabine showed significant increase on freezing during the training on Day 0 (*p = 0.0336*) and reduction of freezing only on Day 1 (*p = 0.0051*), (n = 30; Fig. 2c, d).

### 3.3. c-Fos activation in the hippocampus

**Figure 3.**
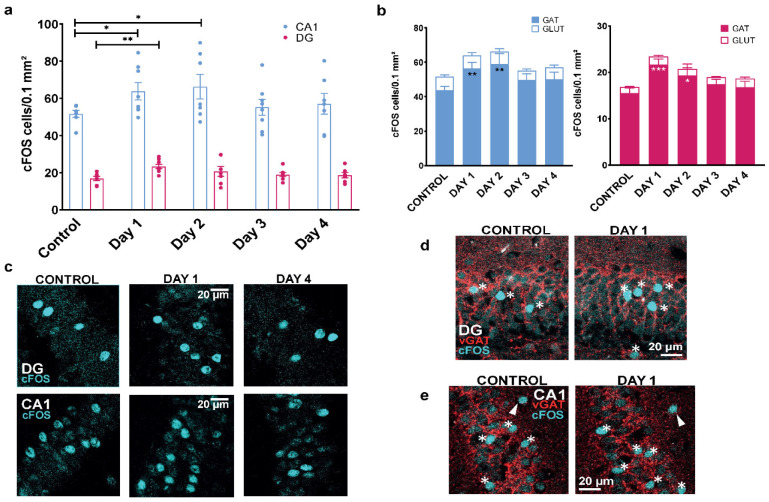
c-Fos expression in hippocampal neurons following contextual Fear conditioning. (a) c-Fos activation patterns during contextual fear conditioning in the CA1 and DG regions of the hippocampus. In the total CA1 neuronal population (blue dots), a significant increase of 27.6% in c-Fos expression was observed in day 1 (*t-test t(12)=2.793, p= 0.0163*), then 34.5% in day 2 (*t-test t(11) = 2.686, p = 0.0212*), which was followed by a return near control values during days 3 and 4 (*n = 7–8; multiple t-test, p > 0.05*). In the other hand, in the total DG neuronal population (pink dots), a significant increase in c-Fos expression of 38.8 % was observed in day 1 (*t-test t(13) = 3.842, p = 0.0020*), which then returned to control levels in days 3 and 4 (n = 7–8; multiple t-test, p > 0.05). *p < 0.05, **p<0.01, ***p < 0.001. (b) A proportion in the expression of c-Fos in vGAT and vGLUT cells in CA1 and DG region of the hippocampus is showed here. In CA1 region vGAT positive neurons, a significant increase of 29.05 % in c-Fos expression was observed at day 1, then 35.2 % at day 2, which was followed by a return to baseline levels at days 3 and 4 during contextual re-exposure. Meanwhile, c-Fos active VGLUT neurons do not change in proportion during any day in CA1 (*n = 7–8; two-ways ANOVA F(1, 64) = 516.5, p < 0.0001*). Furthermore, c-Fos activation patterns in the DG region of the hippocampus in vGAT positive neurons showed an expression significantly increased by 39.9 % at day 1, then by 24.8 % at day 2 and resumed back near control values during contextual re-exposure at days 3 and 4. Additionally, c-Fos active VGLUT neurons do not change in proportion any day in DG (n = 7–8; *two-way ANOVA F (1, 64) = 590.3, p < 0.0001*). Multiple comparisons by Fisher LSD's post-hoc test (vs. control group): *p < 0,05, **p < 0.01, ***p < 0.001. (c) Representative examples of c-Fos immunoreactive neurons of the DG and CA1 regions showing increased expression at day 1 versus control (no-shocked animals) and a subsequent decrease at day 4. (d) Representative examples of vGAT and c-Fos immunoreactive DG neurons showing increased expression at day 1 versus control (no-shocked animals). (e) Representative examples of vGAT and c-Fos immunoreactive CA1 neurons showing increased expression at day 1 versus control (no-shocked animals). The stars show c-Fos immunoreactive cells labeled with vGAT and the arrows show c-Fos immunoreactive cells not labeled with vGAT.

Regarding the crucial role of the hippocampus in CFC [36],[37] and since expression of the immediate early gene c-Fos is a typical marker of neuronal activity [38], we examined how CFC triggered c-Fos expression in hippocampal neurons by immunohistochemistry (IHC). Expression of c-Fos in CA1 and dentate gyrus (DG) hippocampal regions was evaluated one hour after the exposure to the context during the test day (day 1) and during CRP at days 2, 3 and 4. In the total CA1 neuronal population, a small but significant increase of 27.6% in c-Fos expression was observed in day 1 (*t-test t(12) = 2.793, p = 0.0163*), then 34.5% in day 2 (*t-test t(11) = 2.686, p = 0.0212*), which was followed by a return near control values during days 3 and 4 (Fig 3a, c, *p > 0.05*). In the total DG neuronal population, a significant increase in c-Fos expression of 38.8 % was observed in day *1 (t-test t(13) = 3.842, p = 0.0020),* which then returned to control levels in days 3 and 4 (Fig. 3a, c; n = 7–8; *p > 0.05*). To identify how CFC affects c-Fos hippocampal expression in excitatory glutamatergic versus inhibitory GABAergic neurons, triple IHC was performed using antibodies against c-Fos, the vesicular glutamate transporter-1 (vGluT1; labeling glutamatergic cells) and the vesicular GABA transporter (vGAT; labeling GABAergic cells). Most c-Fos immunoreactive cells were predominantly expressed in vGAT positive inhibitory neurons in CA1 (84 % to 90%), and in DG regions of the hippocampus (89% to 93%), (Fig. 3b, d, e). In vGAT positive CA1 neurons, a significant increase of 29.05% in c-Fos expression was observed at day 1, then 35.2% at day 2, which was followed by a return to baseline levels at days 3 and 4 (Fig. 3b, e; *n = 7–8; two-ways ANOVA F(1, 64) = 516.5, p < 0.0001*). In vGAT positive DG neurons, c-Fos expression significantly increased by 39.9 % at day 1, then by 24.8 % at day 2 and resumed back near control values at days 3 and 4 (Fig. 3b, d; *n = 7–8; two-way ANOVA F (1, 64) = 590.3, p < 0.0001)*. Around 6.5% to 10% of c-Fos positive cells were expressed in vGluT1 cells in DG (Fig. 3b; *n = 7–8; two-way ANOVA F (1, 64) = 590.3, p < 0.0001*), and 10% to 15.5% in CA1 (Fig. 3b; *n = 7–8; two-ways ANOVA F(1, 64)= 516.5, p < 0.0001*), but no significant changes were found between days in any of both areas.

### 3.4. Impact of the fear response on the axon initial segment of hippocampal neurons

**Figure 4.**
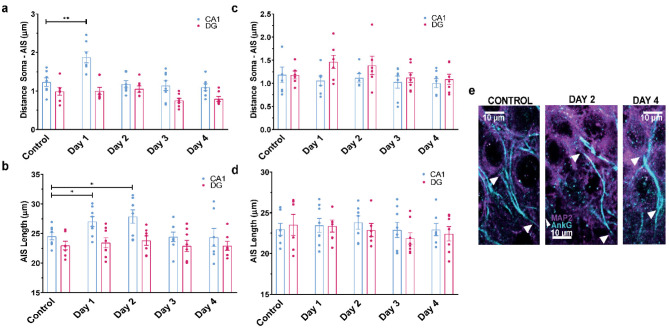
Impact of the fear response on the axon initial segment of hippocampal neurons. (a) Distance from the soma of the AIS in CA1 and DG hippocampal GABAergic neurons immunolabeled with AnkG and vGAT antibodies during contextual fear conditioning. A significant increase in the distance Soma-AIS in CA1 was found in Day 1(n = 7–8; CA1: ANOVA F(4, 31) = 7.713, p = 0.0002, Dunnett's post-hoc test *p < 0.05, **p < 0.01; DG: ANOVA F(4, 32) = 2.873, p = 0.0386). (b) AIS length of CA1 and DG hippocampal GABAergic neurons immunolabeled with AnkG and vGAT antibodies during contextual fear conditioning. A significant longer AIS segment was observed in vGAT positive GABAergic neurons of the CA1 region at day 1 and day 2 (n = 7–8, ANOVA, F(4, 34) = 2.847, p = 0.0388, Dunnett's post-hoc test *p < 0.05, **p < 0.01). (c) Distance from the soma of the AIS in CA1 and DG hippocampal glutamatergic neurons immunolabeled with AnkG and vGluT1 antibodies during contextual fear conditioning (CA1: *n = 7, ANOVA F(4, 29) = 0.3647, p=0.8317*; DG: *n = 7*, *ANOVA F(4, 28) = 1.639, p = 0.1923*). (d) AIS length of CA1 and DG hippocampal glutamatergic neurons immunolabeled with AnkG and vGluT1 antibodies during contextual fear conditioning (CA1: *n = 7–8, ANOVA F(4, 32) = 0.2256, p=0.9221*; DG: *n = 7–8*, *ANOVA F(4, 29) = 0.6468, p = 0.6336*). (e) Representative examples of AIS in CA1 GABAergic neurons labeled with AnkG (blue color) and MAP2 (pink) antibodies in c-Fos positive cells. The arrows show the start and the endpoint of each AIS.

Since the hippocampal network plays a pivotal role in CFC and because of the lack of knowledge of the AIS plasticity in a behaviorally relevant context, we investigated whether structural alterations of the AIS in hippocampal neurons occurred during contextual fear memory acquisition and short-time extinction in mice. To determine how the fear response affects the structural plasticity of the AIS, IHC was processed one hour after the exposure to the context. The AIS segment was immunolabeled with Ankyrin G (AnkG) antibodies. The distance from the soma and the length of the AIS were measured in two parallel series of triple labeling using antibodies against c-Fos, vGAT and AnkG or c-Fos, vGluT1 and AnkG. In vGluT1 positive excitatory neurons of the CA1 and DG regions, no significant changes were found neither in the soma-AIS distance nor in the AIS length at any time of the CFC (Fig. 4c, d). In vGAT positive inhibitory neurons of CA1 area a significant difference was found in the soma-AIS distance at day 1 of the CFC (Fig. 4a; *n = 7–8; ANOVA F(4, 31) = 7.713, p = 0.0002*), but no significant differences were found in DG (Fig. 4a; *n = 7*–*8; ANOVA F(4, 32) = 2.873, p = 0.0386)*. On the other hand, a significant longer AIS segment was observed in vGAT positive GABAergic neurons of the CA1 region at day 1 and day 2 (Fig. 4b; n = 7–8; length control = 24.52 ± 0.5 µm versus length day 1 = 27.02 ± 0.83 µm, and day 2 = 27.8 ± 1.1 µm; ANOVA, *F(4, 34) = 2.847, p = 0.0388*). In contrast, no significant differences were found in the AIS length of GABAergic neurons (vGAT positive) in the DG region at any time of the CFC (Fig. 4b).

## 4. Discussion

Fear acquisition and extinction depends on specific structures such as the amygdala, the hippocampus, and the prefrontal cortex [39],[40]. Understanding the cellular mechanisms underlying fear extinction might lead to the development of novel therapeutic approaches, which when combined with psychotherapy might ease fear extinction and prevent distress recurrence. At the cellular level, Santini et al., [26] showed that the excitability of pyramidal neurons from the IL region is a key determinant for both acquisition and extinction of fear, being reduced by fear acquisition and increased by fear extinction. The same group showed that stimulation of muscarinic receptors, which inhibit the M-current, excites IL neurons allowing them to increase firing at high frequency [41]. They found that systemic stimulation of muscarinic receptors enhances recall of fear extinction and that blocking muscarinic receptors systemically or locally in IL disrupts recall of fear extinction [41]. Interestingly, enhancing the excitability of IL neurons by inhibiting M-type K^+^ channels with the blocker XE991 was shown to reduce fear expression and facilitate extinction recall [27]. However, another study reported different results showing that systemic injection or local BLA application of the M-type K^+^ channel blocker XE991 immediately after moderate fear conditioning increased the strength of fear memory consolidation [28]. In the present work, we explored the impact of M-type K^+^ channel blockade or activation on contextual fear acquisition and extinction in order to clarify the role of M-type K^+^ channels in fear conditioning. Furthermore, in view of the essential role of the hippocampus in CFC, we examined c-Fos expression as a marker of neuronal activation in hippocampal neurons. We also explored potential alterations in the AIS of hippocampal neurons during contextual fear memory acquisition and extinction.

When we used similar doses described in Young and Thomas [28] with a single systemic injection of the M-channel blocker XE991 or the M-channel opener retigabine immediately after the foot shock session, no statistical differences were obtained in the freezing response in the shocked mice between either drugs and the vehicle (Fig. 2a, b). However, those previous studies have been done in cued fear conditioning and our experiments were done with contextual fear, and there is not data reported with contextual fear. In contrast, when a single systemic injection of the M-channel blocker XE991 was carried out 15 min before the foot shock session, fear expression was significantly reduced (Fig. 2c, d). Our data are aligned with those of Santini and Porter [26], despite the different timing and routes of administration. While they carried out intra-IL infusion of XE991 on day 2 before extinction training, we performed a single IP injection of XE991 at day 0, even before the foot shock session and obtained similar results, which clearly suggests that M-channel blockade reduces fear expression and facilitates extinction. On the other hand, under our experimental setting retigabine did not modify fear expression in a relevant manner. Retigabine has shown anxiolytic effects in rats and mice [42], hence we would expect that retigabine enhances fear expression and delays its extinction as reported by Young and Thomas [28], it is possible that the intraperitoneal injection of the drug at 10 mg/kg on day 0 did not yield a sufficient retigabine concentration in the IL region to produce its effect. Alternatively, though less likely, it is possible that we reached a maximum degree of freezing under our experimental conditions that could not be overtaken by retigabine. In addition to M-type K^+^ channels, other K^+^ channel subtypes were also found to regulate fear expression and extinction by affecting neuronal excitability in a similar way. Correlating fear extinction with increased neuronal excitability in the IL region, blockade of small conductance Ca2+-activated K+ channels SK2 by apamin late during extinction training facilitated fear extinction in rats [43]. Conversely, infusion of DCEBIO, an SK2 channel opener into IL prior to fear extinction impaired recall of fear extinction without affecting acquisition of extinction [43]. Another study reported that the loss of GIRK2 G-protein-coupled inward-rectifying K+ channels in forebrain pyramidal excitatory neurons, but not GABAergic neurons, impaired contextual fear learning, a task that relies primarily on the dorsal hippocampus [44].

Expression of c-Fos generally reflects strong neuronal activation associated with increased metabolic activity and firing of action potentials or moderate neuronal stimulation, associated with depolarization and intracellular Ca^2+^ entry, sufficient for triggering c-Fos expression [45],[46]. In the present study, the expression of c-Fos showed a significant increase upon CFC, when measured one hour after exposure to the context at day 1 and day 2 in CA1 and DG hippocampal regions. Interestingly, most of c-Fos immunoreactive cells (≈83 % to 92%) were predominantly expressed in vGAT positive inhibitory neurons of both in CA1 and DG regions of the hippocampus, while less than 20% of c-Fos positive cells corresponded to glutamatergic excitatory (vGluT1) neurons (Fig. 3). This labeling pattern may reflect different kinetics of c-Fos expression. In a mouse model of temporal lobe epilepsy, c-Fos expression was primarily restricted to excitatory dentate granule cells of the hippocampus by 15 min after the beginning of spontaneous seizures. At later time points, such as 4 h after a spontaneous seizure, c-Fos labeling decreased in DG granule cells and strongly appeared in GABAergic interneurons within the dentate gyrus and in widespread regions of the temporal lobe [47]. The predominance of c-Fos labeling in inhibitory neurons may also reflect the importance of GABAergic cell function in fear expression and extinction. It is well known that inhibitory GABAergic circuits have important role in the acquisition, memory formation and expression of fear/anxiety episodes, not only by tuning excitatory transmission but also by playing more active roles in intrinsic fear pathways [48]. Studies indicate that the inhibition of a subpopulation of hippocampal parvalbumin-expressing interneurons induced a long lasting anxiety- or fear-like behavior [49]. In addition, parvalbumin-expressing interneurons were identified as critical cellular substrate of fear memory persistence and associated sharp wave-ripple complex activity in the hippocampus [50]. Also, recent results showed that hippocampal CCK-expressing GABAergic neuron activation enhances the retrieval of contextual fear memory [51].

Considered for a long time as the trigger zone for action potential initiation, the AIS was regarded as a static and rigid structure encompassing ion channels anchored to a scaffolding and cytoskeletal protein network. Now, it is recognized that the AIS is a highly dynamic structure, capable of structural and functional plasticity both during development and in the adult by continuously adapting to changes in neuronal activity [52]–[59]. In this work, we examined whether structural alterations of the AIS in hippocampal neurons occurred during contextual fear memory acquisition and extinction. No significant changes were found neither in the soma-AIS distance nor in the AIS length in excitatory neurons of CA1 and DG regions, at any time of CFC. In line with the importance of GABAergic interneurons in fear expression/extinction, a significant longer AIS segment was observed in vGAT positive GABAergic neurons of the CA1 region at day 1 and day 2, and increase in distance soma-AIS in day 1 (Fig. 4). These modest but significant changes in AIS morphology implies an enhanced excitability of inhibitory hippocampal neurons, which may be involved in the process of fear expression. We do not know, however, whether this AIS plastic behavior of hippocampal GABAergic neurons is subsequent to an intrinsic hippocampal trigger or whether it corresponds to a metaplasticity event involving another brain region converging to the hippocampus. Interestingly, it has been reported the involvement of GABAergic system in different stages of fear [48]. The activation of hippocampal GABAA receptors generates a state-dependent contextual fear memory, meaning that contextual fear memory is disrupted, perhaps because fear expression is dependent on the simultaneous engagement of hippocampal GABAergic system [60]. In addition, it was recently reported that *in vivo* optogenetic photostimulation of CCK-expressing GABAergic neurons in the basal amygdala facilitates fear disappearance [61]. Along the same line, reduction in GABAergic synapses innervating specifically the AIS of principal neurons of BLA, by neurofascin knockdown, impairs fear extinction [62].

## 5. Conclusions

In this work, we showed that a single systemic injection of the M-channel blocker XE991 carried out before the foot shock session significantly reduced fear expression. Our data confirm the crucial role of K^+^ channels and notably that of M-type K^+^ channels in the regulation of fear expression by affecting neuronal excitability. Expression of c-Fos was increased following CFC, mostly in GABAergic neurons at day 1 and day 2 in CA1 and DG hippocampal regions. A significant longer AIS segment was observed in GABAergic neurons of the CA1 region at day1 and 2. Our findings highlight the role of M-type K+ channels in CFC and the importance of hippocampal GABAergic neurons in fear expression.
